# Novel small molecule agonist improves behavior deficits and synaptic plasticity in a model of Alzheimer’s disease

**DOI:** 10.1002/alz.092794

**Published:** 2025-01-09

**Authors:** Raj Amin, Ian Steinke, Fajar Wibowo, Sieun Yoo, Vishnu Suppiramaniam, Meenakshi Singh, Robert Rusty Arnold

**Affiliations:** ^1^ Auburn University, Auburn, AL USA; ^2^ Kennesaw state university, Atlanta, GA USA

## Abstract

**Background:**

The increased incidence of Alzheimer’s disease (AD) rate represent an unmet medical need and thus critical for the development of novel molecular therapeutics. Recent work focusing on patients with apoE4 alleles has highlighted the association of brain cholesterol dysregulation with elevated pathological burden and neurodegeneration. These studies have highlighted the importance of the nuclear receptor Liver X receptor (LXR) for developing AD therapies. Unfortunately many current LXR agonists have failed at the clinical levels due to *hepatotoxicity and neutropenia in humans*. Our newest LXR agonist, AU403, was developed in silico as a novel Liver X Receptor‐β (LXRβ) with partial PPARδ/α activity. Thus our design serves as a dual LXRβ/ PPARα agonist. This strategy seeks to avoid the unwanted side effects of traditional LXRα agonists. Lastly our studies in 3xTgAD mice have observed an increase in gene and protein expression of ABCB1, the lipid efflux protein.

**Method:**

Our library of compounds and lead compound AU403 was designed computationally using Schrodinger software and molecular dynamics to model enzymatic activity using crystal structures of LXR beta and alpha. Further AU403, drug design was optimized by modeling ADME properties using both QikProp and GastroPlus to enhance distribution to the CNS. 11 month aged 3xTgAD mice were given orally 10mg/Kg/day for 1 month. Animal behavior for working declarative memory was observed, using Y‐maze and Novel object recognition studies Electrophysiological studies (LTP) and analysis of pathological markers (Amyloid beta and pTau) were measured.

**Result:**

Our *in silico* design led to the development of our lead compound, AU403, which avoids Trp443 and Leu439, which are deep in the LXRα‐AF2 ligand binding pocket. Furthermore, we observed significant improvement in working declarative memory and long‐term potentiation in 12‐month‐old 3xTgAD mice treated with our lead compound AU403. Mechanistically, we observed enhanced microglia interaction with dendritic spine density and reduced amyloid‐beta plaque levels as well as reduced pay levels.

**Conclusion:**

We have observed that AU403 can serve as a highly novel potential therapeutic agent for AD.

